# Migrastatics: an overview of the current scenario and future cancer treatments

**DOI:** 10.3389/fonc.2026.1907560

**Published:** 2026-09-02

**Authors:** Irene Olivas-Cano, M. Angeles Juanes

**Affiliations:** 1 Cytoskeletal Dynamics in Cell Migration and Cancer Invasion Lab, Department of Cancer, Centro de Investigación Príncipe Felipe (CIPF), Valencia, Spain; 2 School of Health and Life Science, Teesside University, Middlesbrough, United Kingdom; 3 National Horizons Centre, Teesside University, Darlington, United Kingdom

**Keywords:** actin cytoskeleton, cancer, epithelial-to-mesenchymal transition, metastasis, microtubules, migrastatics

## Abstract

Metastasis is the main cause of cancer-related death worldwide. Thus, significant efforts have been directed towards inhibiting metastasis to avoid regrowth or relapse after cytostatic and/or cytotoxic cancer treatments. Drugs targeting cell-invasiveness are known as migrastatics and primarily target the cytoskeleton to impair cell motility and/or invasive mechanisms. However, the cytoskeleton is essential to perform many fundamental processes, including cell division. Consequently, migrastatics often prompt high toxicity, among other serious side effects, which have prevented their clinical use to this date. In this review, we present the current migrastatics scenario, including their main limitations, and discuss proposed alternatives.

## 1 Introduction

Metastasis is the spread of cancer cells from a primary tumor to distant tissues, which cancer cells colonize to form a new tumor (1). This is a breaking point in patients’ prognosis, being metastasis the most common cancer-related death cause worldwide (2). Great efforts have been made to design anti-metastatic drugs, yet no specific treatment has successfully reached the clinic. Treating metastasis presents particular challenges, as it is an evolving, systemic disease with high adaptability. Therapies aiming to stop metastasis were coined as ‘migrastatics’ in 2017, and described as “*drugs interfering with all modes of cancer cell invasiveness and, consequently, with their ability to metastasize (e.g., inhibiting not only local invasion, but also extravasation and metastatic colonization)*” (3).

To metastasize, cancer cells follow the metastatic cascade, consisting of 1) dissemination, 2) dormancy, and 3) colonization. To disseminate, cancer cells shed from the primary tumor mass and puncture the extracellular matrix (ECM). They then move into blood or lymphatic vessels via intravasation, travel through these systems, and eventually exit via extravasation to invade distant organs. In that new tumor microenvironment (TME), cancer cells enable angiogenesis, and immune-suppression mechanisms to outgrow and colonize the organ. Importantly, macrometastasis is preceded by micrometastasis in the form of circulating tumor cells (CTC), or dormant disseminating cells/niches. This state is difficult to detect in patients but often responsible for therapy resistance, and eventual cancer relapse (1, 4). Thus, metastasis is a highly dynamic and adapting process, which can enter and exit dormancy to escape immune attacks, or nutrient scarcity. Additionally, dormant reservoirs can re-activate disease development. Hence, it is urgent to target essential mechanisms underlying metastasis to efficiently stop it at early stages of the process. Co-administration of migrastatics and cytotoxic therapies would be the most adequate strategy to both eliminate existing cancer cells and prevent relapse (5).

### 1.1 Cytoskeletal dynamics play a key role in metastasis regulation

The intricate regulation of the metastatic process is highly dependent on the cytoskeleton, which is composed by several networks: actin, microtubules, intermediate filaments, and septins. Here, we will focus on the role of actin and microtubule networks. These are tightly intertwined and their crosstalk enables several cellular functions, including mitotic spindle formation, structural support, and cell motility (6). Their dynamism is driven by the rapid polymerization and depolymerization of actin filaments or microtubules, built up by monomeric (G-) actin or tubulin, respectively. Their complexity and wide range of functions rely on several mechanisms, including elongation, capping, and severing. These processes require a coordinate interplay between different nucleators and binding/regulatory proteins and enable cytoskeletal adaptation to biochemical/biophysical stimuli. For a more comprehensive overview of key players and regulatory mechanisms, we refer readers to (7–10).

Dynamic reorganization of different cytoskeletal structures allows for cell movement and plasticity, essential at different phases in metastasis. First, for cells to shed from the primary tumor and then to adapt to the TME, where they encounter different ECM composition and stiffness that are associated with specific invasive patterns (11). As they advance, migrating cancer cells continuously reorganize the microtubule and actin cytoskeleton by undergoing epithelial-to-mesenchymal transition (EMT) and mesenchymal-to-ameboid transition (MAT), which allows them to switch between a mesenchymal degrading mode and an amoeboid rapid movement, respectively. Upon EMT induction, microtubules and intermediate filaments build an abundant network which in coordination with the actin cytoskeleton favors cell motility and invasiveness. EMT allows cells to switch from a tightly adhered epithelial phenotype to a migrating mesenchymal phenotype. The latter is characterized by loosen cell-cell contacts (mainly through protein reorganization at the cell junctions, including E-cadherin and actin), and increasing cell-ECM interactions (mainly by generating invadopodia) (12–14). Invadopodia are dynamic F-actin-rich protrusive structures extending from the cell membrane which puncture the ECM, and degrade the ECM by the action of matrix metalloproteinases (MMP) (14, 15). During invadopodia elongation, the extension of the protrusions is promoted by actin filament polymerization, orchestrated by a combined effect of Arp2/3 complex branching, and β1 integrin-mediated adhesion to the ECM, among other cytoskeletal players like microtubules. Cells also generate lamellipodia, actin-based protrusions that attach to the ECM via integrins at focal adhesions. Mechanosensing mechanisms trigger the F-actin and microtubule crosstalk, which retracts the protrusion propelling the cell forward. Cell contractility is especially relevant when cells encounter confined spaces, where they undergo MAT, then adopting the amoeboid migration mode. This is characterized by rounded cell shape, blebbing, fast migration, and lack of proteolysis activity (16). In ameboid cells, RhoA induces unbranched actin polymerization by mDia1, while ROCK activates myosin, resulting in cells forming a contractile actin cortex (17, 18). Actomyosin contraction forces allow cells to squeeze through pores and push the ECM to advance further.

Moreover, to enter and exit the circulatory system, cancer cells need to cross endothelial barriers. To that end, cancer cells display coordinated interactions among cytoskeletal components, using invadopodia as drilling structures to penetrate endothelial cell-cell junctions (19). To exit circulation via extravasation, cancer cells interact with immune cells in the bloodstream to adhere to the endothelium, while microtubules form microtentacles for cell attachment, and actin filaments enable cells to cross endothelial barriers using actomyosin forces (19–21). Moreover, to survive in circulation, cytoskeleton remodeling allows cancer cells to endure shear stress in the fluidic environment by increasing actin filaments and stress fibers (22). For all the above-mentioned roles, microtubules and the actin cytoskeleton constitute therapeutic targets to fight metastasis.

## 2 Migrastatics advances and limitations

### 2.1 Migrastatics potential

**Figure 1:**
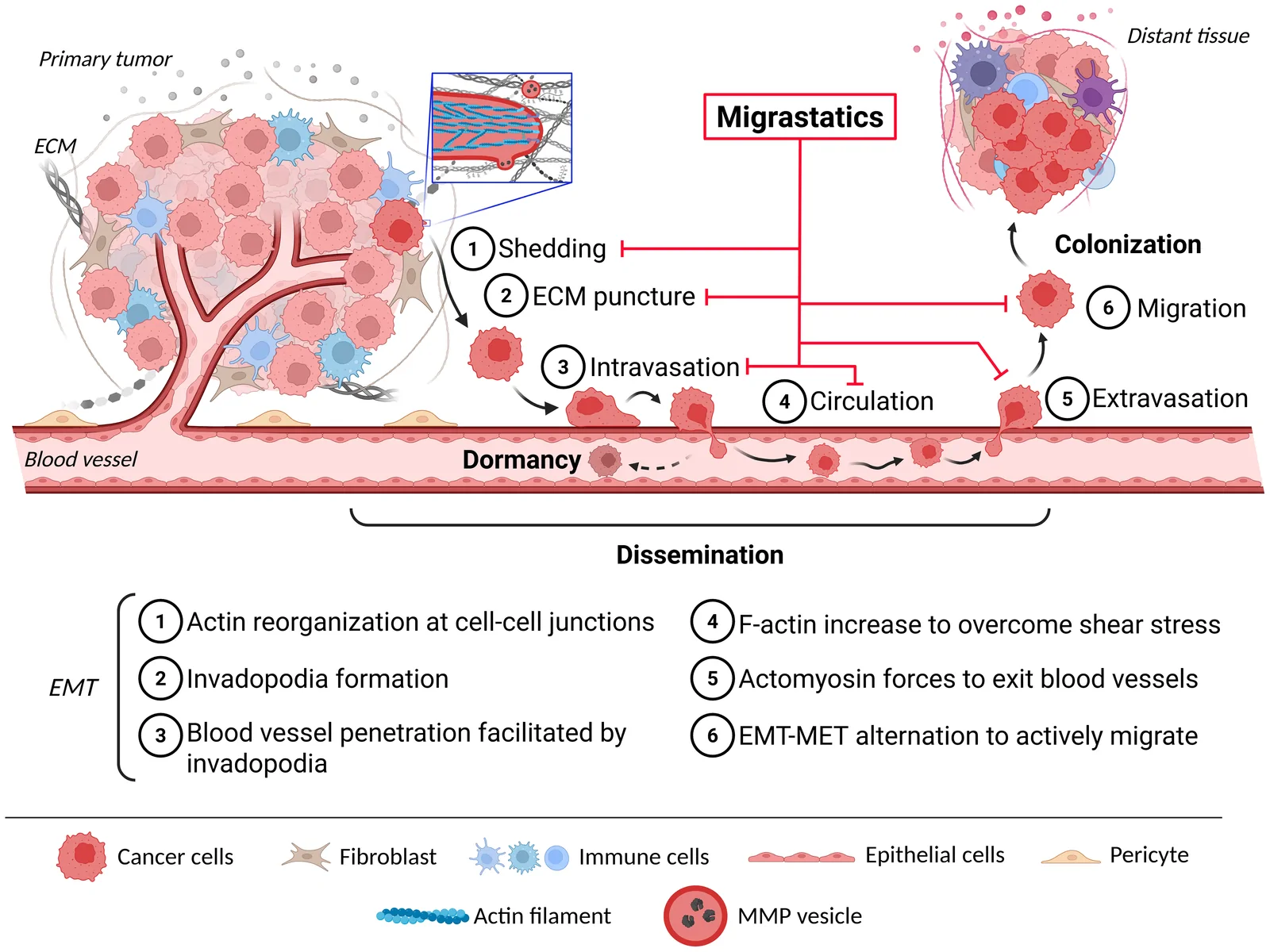
Migrastatics can target different processes in the metastatic cascade. Schematic of the metastatic cascade: dissemination, dormancy, and colonization (bold), depicting EMT steps regulated by cytoskeletal dynamics (1–6), including invadopodia formation (zoom). Inhibition line connectors (red) showing potential action of migrastatics on several metastatic steps. Created in BioRender. Juanes, M. A. (2026) https://BioRender.com/rkcl2x2.

In the search of anti-metastatic therapies, Gandalovičová et al., coined the term “migrastatics” to clearly separate drugs targeting cancer cell invasion and migration (**Figure 1**) from cytostatic therapies, which aim to inhibit cancer cell proliferation. Both migrastatic and cytostatic therapies differ from conventional cytotoxic treatments like chemotherapy or radiation, which directly destroy cancer cells (3, 23–26). Although these cell-killing therapies are the standard-of-care for cancer patients, they exert selective pressure on tumor cells, favoring resistant cells to proliferate and escape the therapy, leading to relapse. Furthermore, some of the standard chemotherapy and/or radiotherapy treatments have been found to increase the metastatic capacity of cancer cells. For example, irradiated cells may be more invasive than non-treated cells, and chemotherapy can trigger tissue repair programs that enhance cancer cell metastasis, sometimes by facilitating EMT. Even surgery, such as fine-needle biopsy for diagnosis, can disrupt cells, as well as the TME, facilitating migration (27). In other cases, the pro-metastatic effects come from support therapies designed to compensate for adverse effects of cytotoxic therapies. For instance, G-CSF (granulocyte-colony stimulating factor) administration is common in chemotherapy treatments to prevent neutropenia. However, G-CSF has been found to promote brain metastases and primary tumor growth in breast cancer (28). Considering the negative impact from extended use of cytotoxic drugs and other anticancer therapies, it is appealing to develop less aggressive therapies, like migrastatics, to prevent disease progression without favoring resistance by selective pressure (23).

Besides, migrastatics are not limited to a particular format or source, as they can be found as small molecules, plant extracts, peptides and/or proteins, among others. This broadens the possibilities of identifying and designing migrastatics, enabling a targeted and optimized therapy against metastasis.

### 2.2 Migrastatics current scenario

Migrastatics target diverse players and processes in cytoskeleton dynamics, including both polymerization and depolymerization of microtubules and actin filaments.

Many drugs act as microtubule-targeting agents (MTA), and some have been/are used in the clinic for different types of cancer (i.e. paclitaxel and analogues for breast, prostate, lung, neck cancer, among others) (29). Many of these MTAs target the taxane site of *β*-tubulin, although different mechanisms apply. For example, zampanolide (30, 31) and taxanes (e.g. paclitaxel and analogues like docetaxel) (32–34) are stabilizing agents that avoid microtubule disassembly. In contrast, de-stabilizing agents such as nocodazole, that leads to microtubule depolymerization, can impair migration in lung, breast, and colorectal cancer cells, among others (35).

Other migrastatics act as stabilizing or destabilizing agents of the actin cytoskeleton. An actin stabilizer is the *Vincetoxicum arnottianum* extract, which effectively impairs cell invasion and migration by stabilizing filamentous actin in pediatric alveolar rhabdomyosarcoma cell line RH-30 (36). Among actin de-stabilizing agents, there is the Bj-PRO-10c, a peptide isolated from *Bothrops jararaca* venom, that disrupts actin filaments, resulting in decreased actin fibers density and quantity in neuroblastoma cells (37). In breast cancer cells, allosteric ROCK2 (Rho-associated coiled-coil kinase 2) inhibitor S9 (belumosudil derivative) leads to actin cytoskeleton rearrangements characterized by decreased actin filament density, retracted filopodia, and changed cell shape, resulting in a significant impairment of collective cell migration (38). In metastatic melanoma, ROCK1–2 inhibition by AT13148 and CCT129254 reduce cell motility and invasion (39). Cytochalasins, fungi metabolites with long-known anti-cancer effects, have been found to inhibit actin polymerization by binding the barbed end of actin filaments (40–44). Although cytochalasin D promoted lung metastasis in melanoma B16 murine models (45), other cell lines and settings have resulted in migrastatic effects. For example, cytochalasin D successfully inhibited cell migration in skin and breast cancer tumoroids in 3D models using non-toxic concentrations of 0.2-1µM that did not interfere with cell growth (46). Furthermore, different cytochalasan types and analogues have been explored for their migrastatic effect in several cancer cell lines, some showing not only positive results as migrastatics but also safer strategies with less impact on the immune response (44). For example, the prodrug BQTML-CB derived from cytochalasin B significantly inhibits collective cell migration of osteosarcoma-derived cells, while causing a less severe decrease in cell proliferation than the original cytochalasin B. The prodrug is dependent on NQO1, highly expressed in cancer cells compared to healthy cells, hence non-cancerous cells were not as affected by the compound. Furthermore, the prodrug does not impair either proliferation or migration of human neutrophils, unlike cytochalasin B. BQTML-CB thus provides a less harmful pharmaceutical strategy than cytochalasin B (47).

Above-mentioned examples, among others, have demonstrated that migrastatic effect depends not only on the cytoskeletal target but also on cell-specific features and TME, key factors in metastasis (44). Therefore, both cytotoxicity and cell-type dependency must be taken into consideration as they may pose significant limitations to the treatment. However, there is a promising foundation to further develop migrastatics based on these compounds. Some examples have been summarized in **Table 1**.

**Table 1:** Pharmaceutical agents with migrastatic effect and/or potential.

Agents targeting the microtubule cytoskeleton								
Drug	Type	Mechanism	Migration inhibition efficacy	Main side effects/toxicity	Limitations	Cancer type	Approval phase	Ref.
Zampanolide	Small molecule	Irreversibly binding to *β* –tubulin taxane site, microtubule stabilization	60% wound shrinkage decrease in HUVEC cells.	Not reported	Challenging synthesis and limited availability from natural sources (marine sponges)	Ovarian, triple negative breast, etc.	Not yet in clinical trials	(30, 31, 73)
Paclitaxel	Small molecule	Binding to *β* –tubulin taxane site, microtubule stabilization	Significant reduction in HT-1080 migration both in 2D and 3D collagen matrices Up to 90% reduction in directed cell migration from glioma spheroids (line GaMg).	Neuropathy, neutropenia, anemia, anorexia, myalgia, gastrointestinal effects, alopecia, tachycardia, etc.	Toxicity	Breast, endometrial, non-small-cell lung, bladder, etc.	Approved	(32–34, 74–76)
Docetaxel (paclitaxel analogue)	Small molecule	Binding to *β* –tubulin taxane site, microtubule stabilization	<60% wound shrinkage decrease in HUVEC cells.	Hypersensitivity, neuropathy, skin reaction, neutropenia, anemia, gastrointestinal effects, alopecia, myalgia, fluid retention, etc.	Toxicity	Breast, lung, prostate, neck, etc.	Approved	(10, 30, 32–34, 77, 78)
Nocodazole	Small molecule	Binding to *β*-tubulin colchicine site, microtubule depolymerization	Significant reduction in migration speed of MDA-MB 231 cells treated with ≥120 nM nocodazole	Germ cell mutagenicity (animal models)	No clear advantage over existing anti-cancer drugs	Lung, breast, prostate, colorectal, glioblastoma	Not yet in clinical trials	(35)
Agents targeting the actin cytoskeleton								
*Vincetoxicum arnottianum extract*	Plant extract	Unknown binding site, actin fiber stabilization	50-60% decrease in migration rate, and <90% decrease in invasion relative to control in RH-30 cells.	Not reported. No effect on control cells: human fibroblasts (NHDF, C-1285) and mesenchymal stem cells (hMSC, C-12977).	Currently at early stages of development	Pediatric alveolar rhabdomyosarcoma	Not yet in clinical trials	(36)
Bj-PRO-10c	Peptide	Unknown binding site, actin filament disruption	Approx.2-fold dissemination rate decrease in murine models injected with pretreated SH-SY5Y cells	Not reported.	Currently at early stages of development	Neuroblastoma	Not yet in clinical trials	(37)
Belumosudil derivative S9	Small molecule	Binding to ROCK2 hydrophobic pocket, F-actin disturbance	Approx. 20% decrease in wound shrinkage.	Not reported. IC50 similar to approved belumosudil (12.77 µM vs 10.15 µM). Belumosudil causes gastrointestinal effects, fatigue, edema, etc.	Currently at early stages of development	Breast cancer	Not yet in clinical trials *(belumosudil is approved for chronic-graft-versus-host disease)*	(38, 79)
Cytochalasins	Small molecule	Binding to barbed-end of F-actin, polymerization impairment	Complete wound healing inhibition by cytochalasin D in HT-1080 cells. Significant decrease in 2D migration speed in Hs578T cells, and MV3 cells treated with non-toxic concentration of cytochalasins B and D.	Apoptosis induction in cell models	High toxicity in cell models.	Skin, breast, others	Not yet in clinical trials	(10, 41, 44, 46, 80–84)

### 2.3 Migrastatics limitations

The major drawback preventing migrastatic therapies from reaching the clinic to this date is dose-limiting toxicity arising from targeting essential cytoskeletal functions. Most migrastatics target cytoskeletal proteins and/or mechanisms involved in fundamental cellular processes. Consequently, migrastatics impair essential functions (e.g., cell division), and elicit serious side effects not only in cancer cells, but also in healthy cells. Among the latter, immune cells may be prevented from reaching and infiltrating the tumor, as they use the same migration modes as cancer cells and respond to chemokines and cytokines released by both immune and cancer cells in the TME (48). Lack of immune cells migrating from and to the tumor would not only favor immunosuppressive dynamics that benefit cancerous growth (49), but also impede migrastatic-immunotherapy co-administration (50). This is a considerable hustle that even directed delivery systems may not be sufficient to overcome, since the migrastatic therapy would ultimately affect the immune cells present in the tumor. On the contrary, the migrastatic effect on pro-metastatic immune cells, such as macrophages or tumor-associated fibroblasts, may in fact decrease CTCs dissemination and reduce metastasis (51).

Additionally, migrastatics development could be hindered by low efficacy due to compensatory mechanisms and/or cell migration plasticity, which leads to resistance. Due to the fact that the cytoskeleton regulates essential cell processes, redundant compensatory responses on therapies targeting the cytoskeleton are expected. For example, the dynamic switch between migration modes allows cells to escape therapy and continue invasion, as observed upon inhibition of MMPs – which is necessary for mesenchymal migration but induces MAT, enabling amoeboid cell movement (52). Cytoskeletal regulators also present intertwined pathways that can bypass the lack of a particular effector. For example, cytoskeleton remodeling is highly dependent on Rho-GTPases, but RhoA inhibition leads to lack of negative feedback on Rac1, which promotes protrusion formation (53, 54).

Different authors have suggested that potent inhibition of the most fundamental processes, such as overall actin polymerization, would be able to overcome these compensations (3, 26). However, this approach would likely prompt considerable side effects, and other strategies should be considered instead.

### 2.4 Migrastatics future

As stated, many migrastatics have been withheld from clinical trials due to severe side effects related to cytoskeletal impairment. Strategies have been proposed to overcome the broad impact of migrastatics interfering with essential cell functions. For example, accurate delivery systems such as antibody-drug conjugates. A successful FDA-approved example is Ado-trastuzumab emtansine, which targets HER2 receptor (trastuzumab) and inhibits microtubule assembly (emtansine) to treat breast cancer (55). Other strategies are under development, like encapsulation of Ruthenium(III) complexes in crystals of nontoxic metal–organic frameworks to treat hepatoblastoma (56). Importantly, concerns about toxicity may clash with those about compensatory mechanisms hindering the treatment’s efficacy. Despite targeting basic processes and overall cellular actin may ensure efficacy, it is vital to reach a balance between migrastatics effectiveness and low toxicity. Targeting a specific actin nucleator and/or actin-binding regulatory protein would be a safer pharmaceutical strategy.

Additionally, migrastatics success in the clinic is hampered by the lack of an adequate assessment protocol. Unlike traditional cytostatic and cytotoxic treatments, migrastatics efficacy will not be reflected on tumor shrinkage or cell death, which currently determine clinical evaluation. Instead, parameters to measure cell dissemination impairment, such as lack of metastasis across the body, would be reliable factors to assess migrastatics success (26, 32, 57, 58). Maiques et al., in their migrastatics development pipeline, include (A) *in vivo* validation by assessing metastasis using serial whole-body imaging of murine models, and (B) ex vivo validation using extracted tumors (59). These would be reliable and more adequate evaluation methods to assess migrastatics, alongside with ADME (absorption, distribution, metabolism, and excretion) and toxicity assessments as standard clinical trials.

## 3 Discussion and concluding remarks

Different migrastatics have shown promising results in research, and optimization strategies have been proposed to drive migrastatic advances up to clinic standards; for example, directed delivery systems to minimize the toxic effect in healthy cells (60), or targeting basic processes such as actin polymerization to avoid compensatory mechanisms.

However, none of these therapies are available to patients to this date. Thus, it is urgent to develop more specific migrastatic therapies. To efficiently overcome dose-limiting toxicity, migrastatics design should focus on enhancing target specificity, and consider multi-targeted approaches. Aiming for a more specific – hence potentially less toxic – strategy to target actin polymerization, the focus could be shifted from the process to a particular player, i.e., actin nucleator. There are some interesting drugs to further investigate, e.g., CK-666 is an Arp2/3 complex inhibitor that prevents its conformational change into its active form. CK-666 stops lamellipodia generation and cell migration by impairing F-actin branching and remodeling mediated by Arp2/3 complex. CK-analogues ((N-{2-[5-(Benzyloxy)-2-methyl-1H-indol-3-yl] ethyl}-3-bromobenzamide) and (2,4-Dichloro-N-[2-(7-chloro-2-methyl-1H-indol-3-yl) ethyl]-5-[(dimethylamino) sulfonyl] benzamide)) are also currently under study, but none have entered clinical trials (61, 62). SMIFH2 is a small molecule inhibitor of actin filaments nucleated by formins (63, 64). SMIFH2 also has an effect on vimentin, a key intermediate filament component (65). This dual impact represents an interesting strategy targeting the cytoskeleton by different angles, which could be beneficial given the intricate network of cytoskeletal elements that encompasses motility. For instance, targeting a combination of actin nucleators could effectively inhibit a specific pool of actin and/or function, since actin dynamics is often governed by collaborative players. This is the case of formins and Adenomatous polyposis coli (APC), or Spire and Cappuccino, among others. This multi-target approach is mainly hindered by the low druggability of some actin nucleators, as exemplified by APC. To date, there is no drug capable of inhibiting APC-driven actin nucleation, despite the importance of APC as the colorectal cancer gatekeeper, and evidence supporting APC-dependent actin nucleation activity in collective cell migration and invasion (66–70). Of note, the small molecule TASIN-1(truncated APC selective inhibitor 1) exerts cytotoxic effects in models expressing truncated versions of APC (1309 or 1450 residues in length), which lack the actin nucleation activity (71, 72).

Furthermore, targeting not only several players but also different migration modes would highly reduce cell migration plasticity, hence avoiding therapy resistance by phenotypic heterogeneity (favored by MET/MAT) and/or compensation mechanisms, which could result from adaptation to environmental pressures and/or therapeutic stress.

To conclude, there are three fundamental points to consider in a migrastatics strategy: 1) high specificity to avoid eliciting harmful side effects, 2) avoid compensatory mechanisms by targeting basic non-compensable processes, or several elements simultaneously, and 3) adequate evaluation based on metastasis inhibition effect by decreased migration and invasion capability. Fortunately, many professionals are dedicated to further developing migrastatics, and ameliorate treatments against metastasis, either via *de novo* design or drug repurposing.
